# Current status and influencing factors of enteral nutrition interruption among critical patients: a systematic review

**DOI:** 10.3389/fnut.2025.1462131

**Published:** 2025-06-30

**Authors:** Xiaoyan Lu, Xin Wang, Weixia Yu, Jianzheng Cai, Yuyu Wang, Yongzhi Cao, Limi Dan, Qingling Wang

**Affiliations:** ^1^Department of Intensive Care Medicine, The First Affiliated Hospital of Soochow University, Suzhou, China; ^2^Department of Nursing, The First Affiliated Hospital of Soochow University, Suzhou, China

**Keywords:** intensive care, enteral nutrition interruption, current status, influencing factors, systematic review

## Abstract

**Purpose:**

This study systematically reviewed and elucidated the current status and key determinants of enteral nutrition interruption (ENI) in critically ill patients. By shedding light on these factors, we aimed to furnish compelling evidence to mitigate the occurrence of ENI in this critical setting.

**Methods:**

We embarked on a comprehensive search across seven prominent databases, PubMed, Embase, Web of Science, Cochrane Library, Scopus, EBSCO, and Ovid Medline, spanning from their inception to 27 May 2024. Two independent researchers meticulously screened and assessed the quality of the literature, extracting data on the current status and influencing factors of ENI. This rigorous approach culminated in a descriptive systematic review and analysis.

**Results:**

From an initial pool of 2,984 studies, 28 were deemed suitable for inclusion in this review, comprising 20 cross-sectional and eight cohort studies. Moreover, 16 studies highlighted ENI incidence rates ranging from 4.7% to a staggering 100%, with an overall average of 48.3%. Among 17 studies, a total of 4,890 ENI episodes were reported involving 2,008 critically ill patients, translating to an average of 2–3 episodes per patient. Four studies detailed the cumulative ENI duration in 327 critically ill patients, totaling 11037.2 h, with an individual average of 33.8 h per patient. The analysis revealed four primary factors influencing ENI: procedures, gastrointestinal events, feeding tube problems, and hemodynamic instability. Procedures accounted for 29.8%–85.0% of ENI frequency and 34.6%–81.2% of duration, with averages of 63.4% and 52.1%, respectively. Gastrointestinal events contributed to 9.4%–59.7% of ENI frequency and 11.5%–21.4% of duration, averaging 19.2% and 18.1%. Feeding tube problems ranged from 0.9% to 29.3% in frequency and 1.3%–25.6% in duration, with averages of 9.3% and 11.6%. Hemodynamic instability was responsible for 0.9%–20.0% of ENI frequency and 1.1%–5.1% of duration, averaging 3.9% and 2.6%.

**Conclusion:**

The incidence and frequency of ENI in critically ill patients are notably high, with interruptions lasting for extended durations. The primary culprits, procedures, gastrointestinal events, feeding tube problems, and hemodynamic instability, influenced ENI occurrence.

**Systematic review registration:**

https://www.crd.york.ac.uk/prospero/display_recordphp?ID=CRD42024554417, identifier CRD42024554417.

## 1 Introduction

Nutritional status is recognized as a nursing-sensitive outcome and plays critical role in patient recovery and overall wellbeing in critically ill patients. Adequate nutrition is of vital importance in critically ill patients (1). It plays a key role in modulating inflammatory responses, maintaining immune function, slowing skeletal muscle catabolism, promoting tissue repair, and maintaining the gastrointestinal and pulmonary mucosal barrier (2, 3). Meanwhile, adequate nutrition has been shown to reduce infection complications (*P* < 0.03) (4), shorten intensive care unit (ICU) stays (*P* < 0.01) (5), reduce mortality (*P* < 0.01) (6), and enhance long-term recovery (7, 8). However, several studies have shown that critically ill patients receive only around 60% of their targeted nutritional goal (9). That insufficient caloric and protein intake increases the risk of malnutrition (10). Lew et al. (11) reported that the prevalence of malnutrition in the ICU ranges from 38% to 78%. Malnourished patients are at higher risk of complications, including infections, pressure ulcers, impaired wound healing, and prolonged hospital stays, which ultimately result in higher mortality and increased healthcare costs (2, 12).

A common reason for inadequate intake of calories and protein in critically ill patients is an enteral nutrition interruption (ENI) (13). However, there is currently no universal consensus on the definition of ENI. Through a systematic review of the current literature, we have identified notable discrepancies in the criteria used to define ENI across various studies. Some studies define ENI based on its frequency, considering any single interruption in enteral nutrition (EN) support for critically ill patients as an ENI event (14). Others define ENI by its duration, though there is no universal agreement on the precise time threshold. A commonly used criterion is an interruption lasting 1 h or more during continuous EN infusion. For intermittent infusion, ENI is defined as administering EN three times daily for 30 min each, with the patient failing to receive the expected nutrition within that time frame (15, 16). A recent study showed that 68% of patients had a period of ENI during their stay in the ICU (17). ENI impact clinical outcomes and prognosis in critically ill patients. Compared to patients without ENI, the occurrence of ENI is associated with a higher rate of inadequate feeding (54.0% vs. 15.0%) (17) and an increased mortality rate (46.0% vs. 21.7%) (18). Additionally, having three or more interruptions during the ICU stay is associated with a higher risk of mortality (19). The increased mortality may be associated with various factors, which not only contribute to the mortality rate of critically ill patients but could also be one of the causes leading to the occurrence of ENI. For example, frequent diagnostic or therapeutic procedures are one such factor, as they not only interrupt EN delivery but also increase metabolic stress and energy expenditure. Similarly, gastrointestinal intolerance often leads to ENI, which may reflect underlying systemic inflammation or organ dysfunction, affecting the patient’s poor prognosis.

In conclusion, given the role of adequate nutrition in modulating clinical outcomes in critically ill patients, a comprehensive assessment of the current status of ENI and its influencing factors is necessary. Currently, there has not yet been a systematic review published on the status and influencing factors of ENI. Thus, the systematic review aims to explore the status and influencing factors of ENI in critically ill patients including the status of ENI incidence, frequency and duration.

## 2 Methods

This review was pre-registered in PROSPERO (CRD42024554417) and adhered to the Preferred Reporting Items for Systematic Reviews and Meta-analyses (PRISMA) guidelines (20). PRISMA 2020 checklist are provided in Supplementary Tables 1, 2.

### 2.1 Aim

The aim of this study was to investigate the incidence, frequency, and duration of ENl, with the ultimate goal of providing valuable insights to inform and improve patient care strategies in critical care settings.

### 2.2 Search strategy

We systematically searched the electronic databases PubMed, Embase, Web of Science, Cochrane Library, Scopus, EBSCO, and Ovid Medline for eligible studies from their inception to 27 May 2024. The search was limited to full-text articles available in English. Initially, the inclusion of the qualifier “ICU” resulted in a limited amount of literature. Consequently, the search strategy was refined by reducing the emphasis on “ICU” to enhance the retrieval of relevant studies. Following a series of preliminary searches, the final search strategy was determined. This involved combining subject terms and free terms and employing Boolean logic operators to optimize retrieval accuracy. Additionally, the reference lists of identified articles were manually reviewed to uncover any further relevant publications. Detailed search strategies are provided in Supplementary Table 3.

### 2.3 Inclusion and exclusion criteria

Inclusion criteria were as follows: (1) critically ill patients aged 18 years or older receiving EN support; (2) observational studies, including cross-sectional, cohort, and case-control studies; (3) primary or secondary outcome measures included the current status or influencing factors of ENI.

Exclusion criteria were as follows: (1) reviews, conference abstracts, lectures, animal experiments, reader letters, and research protocols; (2) studies with unavailable data extraction; (3) duplicate publications; (4) literature without full-text access; (5) articles with quality assessment scores below five points.

### 2.4 Study selection and quality assessment

Based on the search results, the literature was first imported into the reference manager EndNote X9 for deduplication. Two researchers then independently screened the literature according to the inclusion and exclusion criteria. The screening process involved initially reviewing the titles and abstracts, followed by reading the full texts to determine eligibility. Additionally, the references of included articles were manually searched to identify further relevant studies. In cases where consensus could not be reached, a third researcher was consulted. The quality of the included studies was independently evaluated by two researchers. Cross-sectional studies were assessed using the scale recommended by the Agency for Healthcare Research and Quality (AHRQ), which includes 11 items evaluated with “yes,” “no,” or “unsure.” Each “yes” answer earns one point, resulting in a total score ranging from 0 to 11. Studies scoring 0–3 points were classified as low quality, 4–7 points as medium quality, and 8–11 points as high quality. The quality evaluation of cohort studies was conducted using the Newcastle-Ottawa Scale (NOS). The NOS comprises eight items categorized into three dimensions: selection, comparability, and outcome. Comparability can score up to two points, while each of the other items can score up to one point, with a total score ranging from 0 to 9. Based on the score, literature quality was categorized as low quality (1–3 points), medium quality (4–6 points), or high quality (7–9 points) (21).

### 2.5 Data extraction

Relevant information was extracted from the included literature using a standardized data collection form. The extracted data included: (1) basic information: first author, year of publication, country, department; (2) study type; (3) main inclusion criteria; (4) follow-up duration; (5) outcome indicators; (6) factors influencing ENI; (7) Acute Physiology and Chronic Health Evaluation II (APACHE II) score; (8) number of patients on mechanical ventilation and duration of mechanical ventilation; (9) start time of EN, feeding route, and infusion method of EN after ICU admission; (10) number of patients receiving EN; (11) number of patients with ENI, frequency of ENI, and duration of ENI; (12) main findings.

### 2.6 Data analysis

This study described the current status of ENI in critically ill patients by calculating the average incidence, average frequency, and average duration of ENI. Additionally, data on relevant influencing factors reported in two or more studies were combined by summing up the frequency and duration of ENI and then calculating the average proportion based on factor classification. Factors that could not be combined were subjected to descriptive analysis only.

## 3 Results

### 3.1 Selection process and quality assessment

**FIGURE 1:**
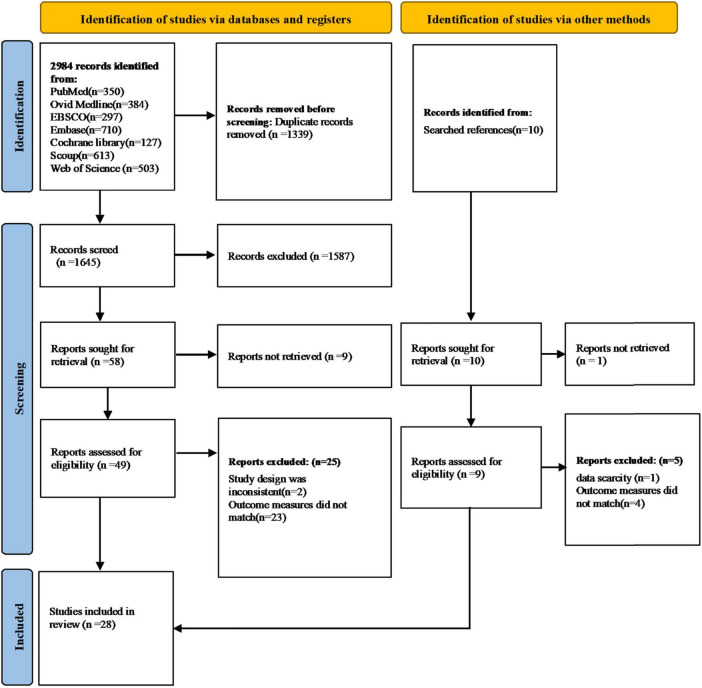
Flow chart of article retrieval.

Figure 1 illustrates the selection process, detailing the number of studies at each review stage. The database search initially identified 2,984 relevant articles. After removing 1,339 duplicates, 1,645 articles were screened. From these, 1,587 were excluded based on titles and abstracts, and nine articles were inaccessible in full text, leaving 49 articles for full-text screening. Ultimately, 24 articles met the inclusion criteria, all of which were met the criteria of quality assessment. A supplementary manual search of the reference lists of these articles yielded an additional four relevant studies, resulting in a total of 28 articles included in the review (13, 14, 17, 19, 22–45). The characteristics of the included studies are detailed in Table 1.

**TABLE 1:** The characteristics of the included studies.

References	Year	Country	Department	Study type	Main inclusion criteria	Follow-up duration(d)	Outcome indicators	Factors influencing ENI	APACHE II score
O’Leary-Kelley et al. (22)	2005	United States	MICU SICU	CCS	Inclusion: ICU patients ≥ 18 years of age who receive mechanical ventilation and EN. Exclusion: patients accept PN or oral feeding.	3	ENI duration	①②③	–
O’Meara et al. (23)	2008	United States	MICU	CCS	Inclusion: ICU patients receive mechanical ventilation without contraindications to EN (e.g., gastrointestinal bleeding, intestinal obstruction, intestinal perforation). Exclusion: patients accept PN.	10	ENI duration and frequency	①②③④	–
Salciute-Simene et al. (17)	2021	Lithuania	MICU SICU	CS	Inclusion: patients receive EN and no oral intake within 48 h of ICU admission. Exclusion: patients accept PN or oral feeding.	7	ENI frequency	①②④	20 ± 8
Onuk et al. (19)	2022	Turkey	MICU	CCS	Inclusion: patients ≥ 18 years of age, started on EN within 72 h of admission to the ICU, and stayed in the ICU for ≥ 48 h. Exclusion: patients accept PN or oral feeding.	7	ENI frequency	①②③④	22 (17–27)
Yip et al. (45)	2014	Malaysia	GICU	CCS	Inclusion: patients ≥ 18 years old who receive mechanical ventilation and EN, staying in ICU for at least 24 h. Exclusion: patients with contraindications to EN (e.g., severe hemodynamic instability, intestinal obstruction, severe prolonged intestinal obstruction, upper gastrointestinal hemorrhage, intractable vomiting or diarrhea, gastrointestinal ischemia).	–	ENI frequency	①②③	–
Kasti et al. (24)	2023	Greek	GICU	CCS	Inclusion: patients with ICU stay > 48 h. Exclusion: patients < 18 years of age receiving oral intake or PN and unable to eat (severe septic shock and hemodynamic instability).	7	ENI patients	①②	–
Ritter et al. (13)	2019	Brazil	GICU	CS	Inclusion: patients receive ≥ 72 h of EN. Exclusion: APACHE II scores could not be calculated for patients who did not undergo arterial blood gas analysis on admission or within 24 h of ICU admission.	7	ENI patients	①②	0–24 (51.5%patients)
Peev et al. (25)	2015	United States	SICU	CS	Inclusion: patients ≥ 18 years of age and receiving EN support for ≥ 72 h. Exclusion: (1) ICU stay < 72 h (2) Received EN support prior to ICU admission (3) Admission diagnosis of intestinal obstruction.	–	ENI frequency	①②	ENI:14.6 ± 6.5 None ENI: 12.8 ± 5.8
Nieuwkoop et al. (26)	2022	Netherlands	MICU SICU	CS	Inclusion: EN started within 48 h and ICU stay ≥ 48 h. Exclusion: patients accept PN or oral feeding.	7	ENI frequency	①②④	61.44 ± 26.17
Kim et al. (27)	2010	Korea	SICU	CCS	–	7	ENI frequency	①②④	–
Kim et al. (28)	2013	Korea	MICU	CS	Inclusion: patient age ≥ 18 years and receiving EN support for ≥ 4 d. Exclusion: patients accept PN or oral feeding.	4	ENI frequency	①②③	13.0 (6.1)
Rice et al. (29)	2005	United States	MICU SICU	CCS	Inclusion: patients received mechanical ventilation for ≥ 72 h and received EN. Exclusion: patients received PN.	–	ENI frequency	①②③④	–
Lee et al. (30)	2018	Malaysia	GICU	CS	Inclusion: patients were ≥ 18 years of age who received mechanical ventilation within 48 h of admission to the ICU and stayed in the ICU for ≥ 72 h.	12	ENI frequency and duration	①②③④	26.93 ± 7.203
Kozeniecki et al. (31)	2016	United States	MICU	CCS	Inclusion: patients ≥ 18 years of age and receiving EN. Exclusion: patient receives intermittent infusion or push feeding.	6	ENI frequency	①②③④	–
Coutris et al. (32)	2019	Canada	SICU	CCS	Inclusion: patient stay in ICU lasts 1 week.	30	ENI frequency and duration	①②③④	–
MacEachern et al. (14)	2018	Canada	MICU SICU	CCS	Inclusion: AL patients with age ≥ 18 years and ICU stay ≥ 24 h.	–	ENI frequency	①②③④	27
Morgan et al. (33)	2004	United States	SICU	CCS	Inclusion: patients ≥ 18 years of age and receiving EN for ≥ 72 h.	7	ENI frequency	①②③	–
Kalaiselvan et al. (34)	2021	India	GICU	CS	Inclusion: patients ≥ 18 years of age and receiving EN for ≥ 48 h.	–	ENI frequency	①②	22.12 ± 7.1
Uozumi et al. (35)	2015	Japan	MICU SICU	CCS	Inclusion: patients ≥ 18 years of age and receiving EN for ≥ 72 h.	–	ENI frequency	①②	21.8 (6.8)
Ramakrishnan et al. (36)	2015	India	GICU	CCS	Inclusion: patients received EN for ≥ 24 h.	–	ENI frequency	①②④	–
Shankar et al. (37)	2015	India	MICU SICU	CS	Inclusion: ICU stay ≥ 72 h.	–	ENI patients	①	Within 6 h: 28.55 ± 7.68 after 6 h: 31.15 ± 8.02
Czapran et al. (38)	2015	The world	GICU	CCS	Inclusion: patients received mechanical ventilation for ≥ 72 h.	12	ENI patients	①②③	21 (6–38)
Passier et al. (39)	2013	Australia	MICU SICU	CCS	–	–	ENI patients	①	15.4(6.1)
Saran et al. (40)	2015	Canada	MICU	CCS	Inclusion: patients ≥ 18 years of age who received mechanical ventilation within 48 h of admission to the ICU and stayed in the ICU for ≥ 72 h. Exclusion: patients who started EN prior to ICU admission, received EN < 3 days, or received PN.	12	ENI patients	②	Gastric :20.2 ± 7.0 post-pyloric: 19.1 ± 6.4
Davies et al. (41)	2011	Australia, New Zealand	GICU	CCS	–	–	ENI duration	①②③	15.9 (7.9)
Ribeiro et al. (44)	2014	Brazil	GICU	CCS	Inclusion: patients ≥ 18 years of age and receiving EN ≥ 72 h. Exclusion: patients receiving palliative care.	–	ENI duration	①②③	–
Elpern et al. (42)	2004	United States	MICU	CCS	Inclusion: patients ≥ 18 years of age and receiving EN. Exclusion: ICU stay < 48 h or receiving oral or PN support.	–	ENI duration	①②③④	19.97 (9–33)
McClave et al. (43)	1999	United States	MICU SICU	CCS	Inclusion: patients receive EN. Exclusion: patients receiving oral feeding or PN support, or no feeding tube placed initially on ICU admission	–	ENI patients	①②③	–
100	–	–	Duodenum (66.7%) gastric (22.9%) empty bowel (10.4%)	–	60	59	–	–	The average duration of ENI per patient per day was approximately 7 h. A total of 59 patients (98.3%) had at least one episode ENI per day, and 43 patients (71.7%) had an average of 2–4 episodes ENI per day.
100	7.4	71% of patients within 48 h, 29% after 48 h	post-pyloric	Continuous infusion	59	–	423	2,540	ENI duration was 27.3% of EN time. The average frequency of ENI per patient was about 1.13 episode per day; the average duration of ENI per patient was about 6 h per day.
–	–	80.8% of patients within 48 h	–	–	73	50	131	–	ENI occurred in 68% of patients during their ICU stay, 35% of EN times, and the median duration of ENI was 12 h.
–	–	Within 72 h	Post-pyloric	–	122	–	334	2,960	The mean frequency of ENI per patient was 2.74, and the median duration of ENI was 960 min.
100	–	Within 15 h	–	continuous infusion	77	61	72	–	Sixteen of the 77 patients (20.7%) did not experience ENI, the rest experienced one or more ENIs, and approximately 61 patients (79%) experienced ENI.
90.1	–	–	–	–	81	81	–	–	ENI was observed in all patients with a median duration of 5.2 (3.4–7.4) h per patient.
100	–	84.6% of patients within 48 h	–	–	130	40	–	–	About 90 ICU patients did not develop ENI and about 40 patients developed ENI.
–	–	Within 48 h	Nasogastric tube, nasoenteric tube, gastrostomy port	continuous infusion	94	64	106	–	A total of 106 ENIs occurred in 64 patients.
86.9	–	Within 48 h	–	–	61	12	115	–	Approximately 20% of participants had at least one episode of ENI during the first 4 days of the study, which tapered off until day 7 of the study. ENI mostly occurred during the first 3 days of ICU admission.
–	–	–	Nasogastric tube	Intermittent infusion	47	26	124	–	The average frequency of ENI per patient was approximately 3.23.
44	–	5.3 d	Nasogastric tube	Intermittent infusion	34	24	54	–	A total of 24 patients (79%) had a total of 54 ENIs within the first 4 days after starting EN. The mean duration of ENI per patient was 360 min.
100	–	35% of patients within 2 d, 65% within 4 d.	93% gastric feeding	Continuous infusion	55	52	179	–	A total of 179 ENIs occurred in 52 patients, and 3 patients (5%) had no ENIs; most (64.2%) ENIs occurred the first 6 days during EN. The mean frequency of ENI per patient was 3.3.
100	–	–	–	Intermittent infusion	148	–	332	4,190	The patient’s ENI occurred around day 3 after admission to the ICU, and the patient’s total duration of ENI was approximately 24.5 h.
–	–	Within 48 h	Gastric tube, jejunal tube	Continuous infusion	78	–	198	–	ENI occurred on 49% of EN days between days 2–6, ENI was maintained for an average of 4.8 h per day.
–	13	8.3 h	–	–	27	–	102	1258.2	ENI mostly occurred in first week after ICU admission, with the frequency of ENI decreasing in the following weeks.
50	11	Within 48 hours	Gastric tube	Continuous infusion	96	–	151	–	The average frequency of ENI was 2 episodes per patient.
–	–	2.2 d	80% gastric feeding 20% jejunal feeding	Continuous infusion	56	–	222	–	ENI occurred a total of 222 episodes in 56 patients.
100	(6.4 ± 4.2)	–	Orogastric or nasogastric tube	continuous infusion	554	–	923	–	A total of 923 ENIs were observed in 554 patients receiving EN support.
95	–	Within 48 h	–	Continuous infusion	100	–	567	–	A total of 567 ENIs occurred in patients, of which 515 occurred in intubated patients (90%). The average frequency of ENI per patient was about three episodes and the duration of ENI was 5.5 h.
–	–	–	–	–	327	327	857	–	ENI occurred in 327 patients for approximately 6360 h. 40% of patients interrupted EN only once, and the remaining 60% interrupted EN twice.
–	–	Within 6 h and after 6 h	–	–	308	62	–	–	The overall incidence of ENI was 20.13%, with no significant difference between the two groups (within 6 h–16.2%; after 6 h–24.7%; *p* = 0.087).
–	–	17 h	81% Gastric tube	–	88	50	–	–	ENI occurred in 50 (57%) patients during ICU.
–	–	–	Gastric tube, jejunal tube	–	69	41	–	–	The mean duration of ENI (from interruption to restart of EN) was 10.7 h.
100	–	Within 48 h	Gastric tube: 91.4%, enteral tube: 5.9%	–	850	160	–	–	Patients with gastric feeding were approximately five times more likely to develop ENI due to gastrointestinal intolerance (bloating, vomiting, high gastric residuals) than patients with small bowel feeding (19.6% and 4.7%, *P* = 0.015).
47	6	Within 72 h	Gastric tube: 55% small bowel tube: 45%	–	58	56	–	–	Within 909 days, 58 patients were treated with EN and 56 had an ENI within 349 (38%) days.
–	–	Within 48 h	Gastric tube, jejunostomy tube	Continuous infusion	93	–	–	3,049	–
100	–	–	Gastric tube, Gastrostomy	Continuous infusion	39	–	–	–	The mean duration of ENI per patient per day was 5.23 h.
95	–	–	Gastric tube	Continuous infusion	44	37	–	–	A total of 83.7% of patients interrupted EN for an average of 19.6% of the EN time.

APACHE II, Acute Physiology and Chronic Health Evaluation II; CS, cohort studies; CCS, cross-sectional studies; ICU, intensive care unit; MICU, medical intensive care unit; SICU, surgical intensive care unit; GICU, general intensive care unit; EN, enteral nutrition; ENI, enteral nutrition interruption; PN, parenteral nutrition; AL, acute leukemia; ; – indicates not mentioned in the original text; ①, Procedures (diagnostic procedures, therapeutic procedures, nursing procedures, airway procedures); ②, Gastrointestinal events (gastrointestinal intolerance, ileus, gastrointestinal bleeding, anastomotic fistula); ③, Tube feeding problems (clogged, displaced, dislodged feeding tubes);④, Hemodynamic instability (shock, weakness, unstable condition, discomfort).

Out of the 28 studies, 20 were cross-sectional, and eight were cohort studies. Among the cross-sectional studies, nine were of high quality, and 11 were of medium quality. For the cohort studies, five were of high quality, and three were of medium quality. The detailed quality assessment results are presented in Tables 2, 3.

**TABLE 2:** Agency for Healthcare Research and Quality (AHRQ) scale to assess the quality of cross-sectional studies.

References	①	②	③	④	⑤	⑥	⑦	⑧	⑨	⑩	⑪	Total
O’Leary-Kelley et al. (22)	Yes	Yes	Yes	Yes	Unsure	Yes	Yes	No	No	Yes	Yes	8
O’Meara et al. (23)	Yes	Yes	Yes	Yes	No	Yes	Yes	Yes	No	Yes	Yes	9
Onuk et al. (19)	Yes	Yes	No	Yes	No	Yes	Yes	Yes	No	Yes	No	7
Yip et al. (45)	Yes	Yes	Yes	Yes	No	Yes	Yes	No	No	Yes	Yes	8
Kasti et al. (24)	Yes	Yes	Yes	Yes	No	Yes	Yes	Yes	No	Yes	No	8
Kim et al. (27)	Yes	No	Yes	Yes	No	Yes	Yes	No	No	Yes	Yes	7
Rice et al. (29)	Yes	Yes	Yes	Yes	No	Yes	Yes	No	No	Yes	Yes	8
Kozeniecki et al. (31)	Yes	Yes	Yes	Yes	No	Yes	Yes	Yes	No	Yes	Yes	9
Coutris et al. (32)	Yes	Yes	Yes	Yes	No	Yes	Yes	No	No	No	No	6
MacEachern et al. (14)	Yes	Yes	Yes	Yes	No	Yes	Yes	No	No	Yes	No	7
Morgan et al. (33)	Yes	Yes	No	Yes	No	Yes	Yes	Yes	No	Yes	Yes	8
Uozumi et al. (35)	Yes	Yes	Yes	Yes	No	Yes	Yes	No	No	Yes	No	7
Ramakrishnan et al. (36)	Yes	Yes	Yes	Yes	No	Yes	Yes	No	No	Yes	No	6
Czapran et al. (38)	Yes	Yes	Yes	Yes	No	No	Yes	Yes	No	Yes	Yes	8
Passier et al. (39)	Yes	Yes	Yes	Yes	No	Yes	No	No	No	Yes	No	6
Saran et al. (40)	Yes	No	Yes	Yes	Unsure	Yes	Yes	No	No	Yes	Yes	7
Davies et al. (41)	Yes	Yes	Yes	Yes	No	Yes	Yes	No	No	Yes	No	7
Ribeiro et al. (44)	Yes	Yes	Yes	Yes	No	Yes	Yes	Yes	No	Yes	Yes	9
Elpern et al. (42)	Yes	Yes	No	Yes	No	No	Yes	Yes	No	Yes	Yes	7
McClave et al. (43)	Yes	Yes	No	Yes	No	No	Yes	Yes	No	Yes	Yes	7

①, Define the source of information; ②, List inclusion and exclusion criteria for exposed and unexposed subjects (cases and controls) or refer to previous publications; ③, Indicate time period used for identifying patients;④, Indicate whether or not subjects were consecutive if not population-based; ⑤, Indicate if evaluators of subjective components of study were masked to other aspects of the status of the participants; ⑥, Describe any assessments undertaken for quality assurance purposes (e.g., test/retest of primary outcome measurements); ⑦, Explain any patient exclusions from analysis; ⑧, Describe how confounding was assessed and/or controlled; ⑨, If applicable, explain how missing data were handled in the analysis; ⑩, Summarize patient response rates and completeness of data collection; ⑪, Clarify what follow-up, if any, was expected and the percentage of patients for which incomplete data or follow-up was obtained.

**TABLE 3:** The Newcastle-Ottawa Scale (NOS) to assess the quality of cohort studies.

References	①	②	③	④	⑤	⑥	⑦	⑧	Total
Salciute-Simene et al. (17)	1	1	1	1	1	1	1	1	8
Ritter et al. (13)	1	1	0	1	1	1	1	1	7
Peev et al. (25)	1	1	1	1	1	1	1	1	8
Nieuwkoop et al. (26)	1	1	0	1	1	0	1	1	6
Kim et al. (28)	1	1	0	1	1	1	1	1	7
Lee et al. (30)	1	1	1	1	0	1	1	1	7
Kalaiselvan et al. (34)	1	1	0	1	1	1	0	1	6
Shankar et al. (37)	1	1	1	1	1	0	0	1	6

①, Representativeness of exposed cohort; ②, Selection of the non-exposed cohort; ③, Ascertainment of exposure;④, Demonstration that outcome was not present at start of study; ⑤, Comparability on the basis of design or analysis; ⑥, Assessment of outcome; ⑦, Was follow-up long enough for outcomes to occur; ⑧, Adequacy of follow-up of cohorts.

### 3.2 The current status of ENI

Given the heterogeneity of EN feeding routes (gastric and post-pyloric), infusion methods (continuous and intermittent), patient characteristics (illness severity, mechanical ventilation status, and EN contraindications), and outcome metrics (ENI incidence, frequency, and duration), a meta-analysis could not be conducted to consolidate the data. Thus, a descriptive analysis of ENI in critically ill patients was performed.

Among the 28 studies included, 16 studies (13, 17, 22, 24–29, 36–41, 45) reported ENI incidence, 17 studies (14, 17, 19, 23, 25–36, 45) reported ENI frequency, and four studies (23, 30, 32, 44) reported ENI duration. The incidence of ENI was reported in 16 studies (13, 17, 22, 24–29, 36–41, 45), of which 2,412 critically ill patients received EN, 1,165 developed ENI, with incidence ranging from 4.7% to 100.0%, and an average rate of 48.3%. Subgroup analyses were conducted based on ICU type, geographical region and publication year. The incidence of ENI were 64.2% in mixed/general ICU, 55.3% in surgical ICU, 25.3% in medical ICU, Studies published in 2014 or earlier reported an incidence of 79.8%, while those published in 2015 or later reported 42.1%. By geographic region, the incidence was 66.5% in Europe, 63.1% in Asia, 31.5% in the Americas and 76.4% in Oceania.

Seventeen studies reported ENI frequency (14, 17, 19, 23, 25–36, 45), with the number of episodes ranging from 1 to 7 in those studies, and a total of 4,890 episodes occurring in 2,008 patients, averaging 2–3 episodes per patient during their ICU stay. Four studies reported the duration of ENI in 327 patients (23, 30, 32, 44), with ranging from 24.3 to 46.6 h in those studies, totaling 11037.2 h during the ICU stay, an average of 33.8 h per patients.

### 3.3 Influencing factors of ENI

All 28 studies (13, 14, 17, 19, 22–45) included in this systematic review reported factors influencing ENI. Due to the heterogeneity of the studies, a descriptive analysis was conducted. Factors mentioned in at least two studies were combined by summing the frequency and duration of ENI and calculating the average proportion for each category.

**FIGURE 2:**
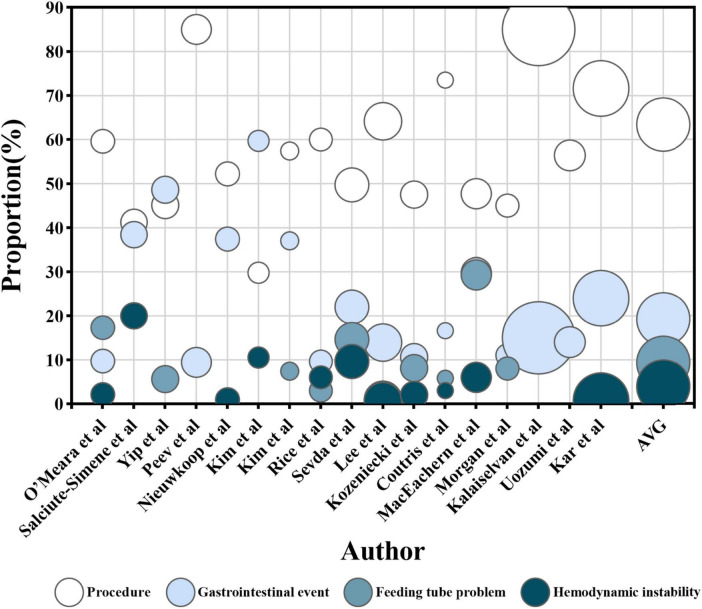
The proportion of enteral nutrition interruption (ENI) frequency. The horizontal axis represents each research, while the vertical axis represents the proportion of ENI frequency caused by each influencing factor out of the total frequency of ENI in single research. The color of the circles represents four influencing factors, and the size of the circles represents the number of participants receiving EN in each research. The AVG represents the average proportion of ENI frequency caused by four different factors, relative to the total ENI frequency, with the circle’s area being meaningless.

**FIGURE 3:**
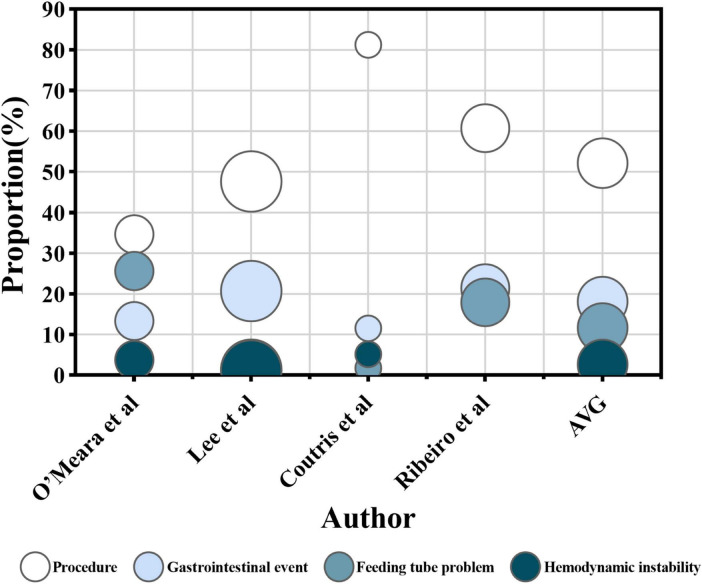
The proportion of enteral nutrition interruption (ENI) duration. The horizontal axis represents each research, while the vertical axis represents the proportion of ENI duration caused by each influencing factor out of the total duration of ENI in single research. The color of the circles represents four influencing factors, and the size of the circles represents the number of participants receiving EN in each research. The AVG represents the average proportion of ENI duration caused by four different factors, relative to the total ENI duration, with the circle’s area being meaningless.

The factors were grouped into four categories: procedures, gastrointestinal events, feeding tube problems, and hemodynamic instability. Procedural factors included airway procedures (e.g., intubation, extubation, and tracheostomy), therapeutic procedures (e.g., surgery, dialysis, and drainage), diagnostic procedures (e.g., imaging and endoscopy), and nursing procedures (e.g., bathing and dressing changes). Gastrointestinal events include intolerance [e.g., high gastric residual volume (GRV > 250 m) (46), abdominal distension, diarrhea, abdominal pain, nausea, vomiting, and reflux aspiration] along with other complications (e.g., gastrointestinal bleeding, ileus, and anastomotic leaks). Feeding tube problems cover problems such as blockage, displacement, and dislodgement. Hemodynamic instability [mean arterial pressure (MAP) < 65 mmHg] (46) is characterized by symptoms including shock, weakness, instability, and discomfort. A detailed descriptive analysis of these four categories and the combined results is presented in Figures 2, 3.

#### 3.3.1 Procedural factors

Among the 28 studies analyzed, 27 identified procedure as an important factor influencing ENI (13, 14, 17, 19, 22–39, 41–45). A total of 17 identified a total of 4,890 interruptions (14, 17, 19, 23, 25–36, 45), with 63.4% on average attributed to procedural factors. The proportion of ENI frequency due to procedures ranged from 29.8% to 85.0% in those studies (Figure 2). Subgroup analyses were conducted based on ICU type, geographical region and publication year. The proportion of ENI frequency due to procedures was 67.8% in mixed/general ICU, 54.5% in surgical ICU and 53.5% in medical ICU. Studies published in 2014 or earlier reported the proportion of 51.7%, while those published in 2015 or later reported 66.7%. By geographic region, the proportion was 48.3% in Europe, 69.3% in Asia and 57.1% in the Americas. Four studies reported a total interruption time of 11037.2 h (23, 30, 32, 44), of which procedural factors contributed to 5746.5 h with an average of 52.1%). The proportion of ENI duration due to procedures ranged from 34.6% to 81.2% in those studies (Figure 3).

**FIGURE 4:**
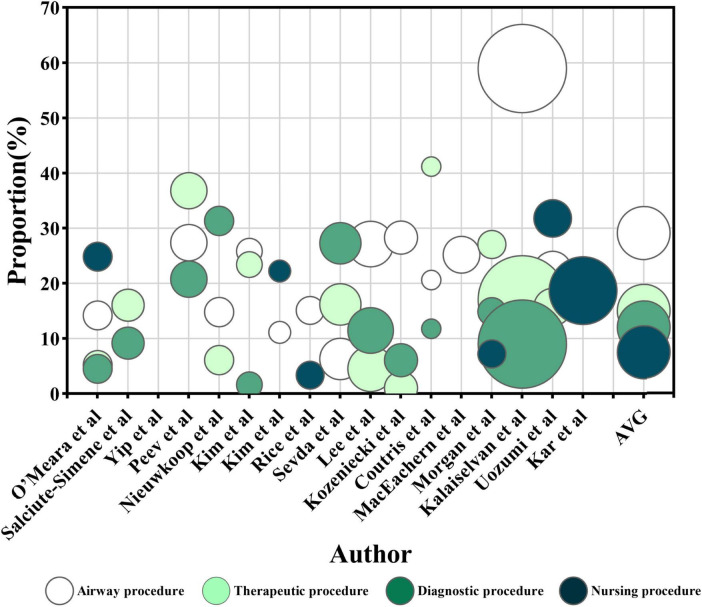
The proportion of enteral nutrition interruption (ENI) frequency caused by different categories of procedures. The horizontal axis represents each research, while the vertical axis represents the proportion of ENI frequency caused by various procedure categories out of the total frequency of ENI in a single article. The color of the circles represents the category of the procedure, and the size of the circles represents the number of participants receiving EN in each research. The AVG represents the average proportion of ENI frequency caused by different procedure categories relative to the total ENI frequency, with the circle’s area being meaningless.

Procedural types contributed to ENI with varying frequency (Figure 4): airway procedures (14, 17, 19, 23, 25–32, 34, 35) were the most frequent, accounting for an average of 29.1% of ENI (1,089 of 3,739). Therapeutic (17, 19, 23, 25–27, 30–35) and diagnostic (17, 19, 23, 25–27, 30–34) procedures accounted for 15.0% (537 of 3,577) and 12.0% (360 out of 3,010) of ENI, respectively. Nursing procedures (23, 28, 29, 33, 35, 36) contributed 7.5% (173 of 2,302).

**FIGURE 5:**
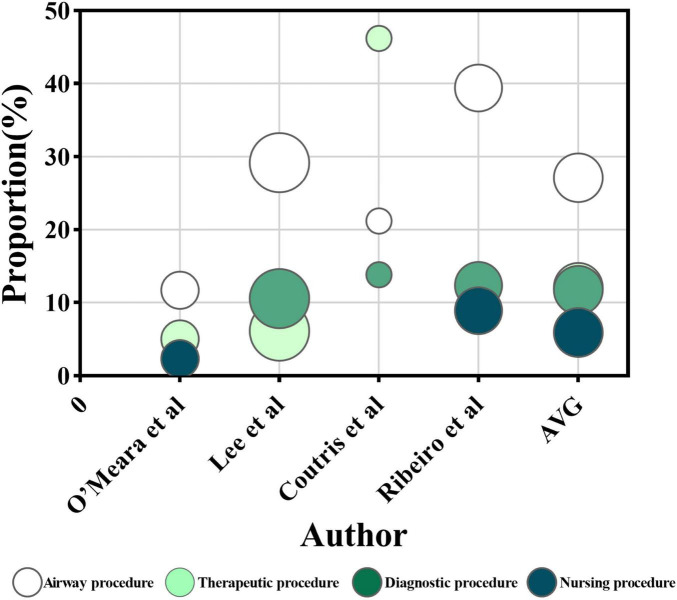
The proportion of enteral nutrition interruption (ENI) duration caused by different categories of procedures. The horizontal axis represents each research, while the vertical axis represents the proportion of ENI duration caused by various procedure categories out of the total duration of ENI in a single article. The color of the circles represents the category of the procedure, and the size of the circles represents the number of participants receiving EN in each research. The AVG represents the average proportion of ENI duration caused by different procedure categories relative to the total ENI duration, with the circle’s area being meaningless.

Regarding ENI duration (Figure 5), airway procedures (23, 30, 32, 44) accounted for 27.1% (2986.4 h) of the total ENI time, while therapeutic (23, 30, 32) procedures accounted for 12.1% (964.1 h) and diagnostic procedures (30, 32, 44) for 11.7% (993 h). Nursing procedures (23, 44) caused 5.9% (330 h) of ENI.

#### 3.3.2 Gastrointestinal events

Among the 28 studies reviewed, 26 identified gastrointestinal events as a key factor influencing ENI (13, 14, 17, 19, 22–36, 38, 40–45). Seventeen studies (14, 17, 19, 23, 25–36, 45) reported a total ENI frequency of 4,890 episodes, with 940 attributed to gastrointestinal events, accounting for an average of 19.2% of ENI frequency. The proportion of ENI frequency due to gastrointestinal events ranged from 9.4% to 59.7% in those studies (Figure 2). Subgroup analyses were conducted based on ICU type, geographical region, and publication year. The proportion of ENI frequency due to gastrointestinal events was 19.8% in mixed/general ICU, 22.6% in surgical ICU and 15.4% in medical ICU. Studies published in 2014 or earlier reported the proportion of 19.7%, while those published in 2015 or later reported 19.1%. By geographic region, the proportion was 28.6% in Europe, 20.4% in Asia and 12.7% in the Americas. Four studies (23, 30, 32, 44) reported a total ENI duration of 11037.2 h, with 2002.8 h due to gastrointestinal events representing 18.1% of the total ENI duration. The proportion of ENI duration due to gastrointestinal events ranged from 11.5% to 21.4% in those studies (Figure 3).

**FIGURE 6:**
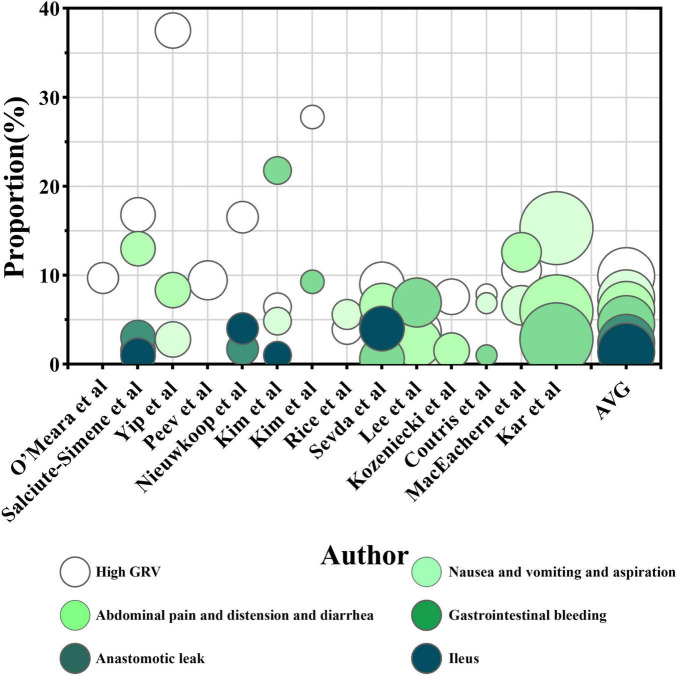
The proportion of enteral nutrition interruption (ENI) frequency caused by different categories of gastrointestinal events. The horizontal axis represents each research, while the vertical axis represents the proportion of ENI frequency caused by various gastrointestinal event categories out of the total frequency of ENI in a single article. The color of the circles represents the category of the gastrointestinal event, and the size of the circles represents the number of participants receiving EN in each research. The AVG represents the average proportion of ENI frequency caused by different gastrointestinal event categories relative to the total ENI frequency, with the circle’s area being irrelevant.

Gastrointestinal events were further categorized to assess their contribution to ENI frequency (Figure 6). High GRV was identified as a significant factor in 13 studies (14, 17, 19, 23, 25–32, 45), causing 230 interruptions out of 2,321, averaging 9.9% of the total ENI. Nausea, vomiting, and reflux aspiration accounted for 7.4% of ENIs (191 of 2,595) (14, 19, 23, 26, 27, 29–32, 36, 45), while abdominal pain, diarrhea, and bloating accounted for 6.1% (126 of 2,075) (14, 17, 19, 30, 31, 36, 45). Gastrointestinal bleeding caused 4.5% of ENIs (82 of 1,803) (17, 26), while anastomotic leaks and ileus accounted for 2.4% and 1.4% (17, 19, 26, 27), respectively.

**FIGURE 7:**
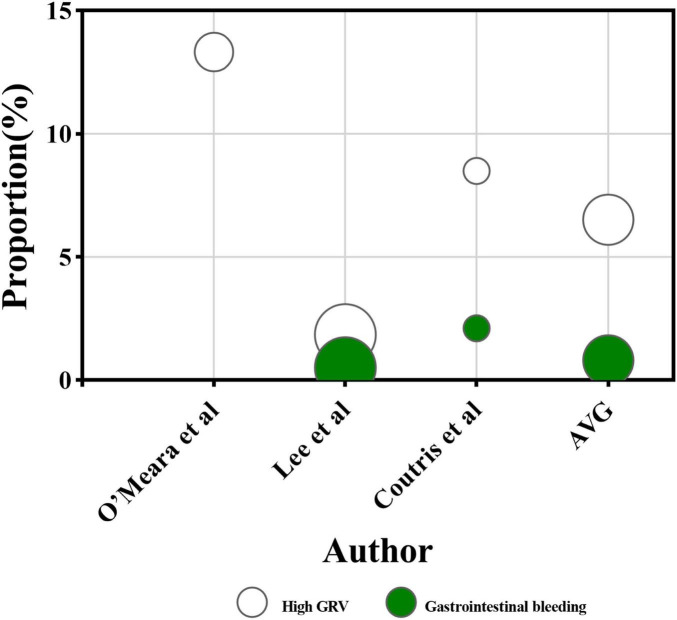
The proportion of ENI duration caused by different categories of gastrointestinal events. The horizontal axis represents each research, while the vertical axis represents the proportion of ENI duration caused by various gastrointestinal event categories out of the total duration of ENI in a single article. The color of the circles represents the category of the gastrointestinal event, and the size of the circles represents the number of participants receiving EN in each research. The AVG represents the average proportion of ENI duration caused by different gastrointestinal event categories relative to the total ENI duration, with the circle’s area being irrelevant.

In terms of ENI duration (Figure 7), high GRV was the primary contributor, causing 521.8 h of ENI across three studies, averaging 6.5% of the total ENI time (23, 30, 32). Nausea, vomiting, and reflux aspiration contributed 46 h, accounting for 0.8% of the total ENI duration (30, 32). The studies conducted by Ribeiro et al. (44), O’Leary-Kelley et al. (22), Elpern et al. (42) did not categorize gastrointestinal intolerance but reported it as a significant factor contributing to 21.4%, 19.8%, and 22.8% of ENI duration, respectively.

#### 3.3.3 Feeding tube problems

Among the 28 studies reviewed, 16 identified (14, 19, 22, 23, 28–33, 38, 41–45) feeding tube problems as a significant factor influencing ENI. Ten studies (14, 19, 23, 28–33, 45) reported a total ENI frequency of 2,067 episodes, with 193 attributed specifically to feeding tube problems, accounting for 9.3% of ENI frequency on average. The proportion of ENI frequency due to feeding tube problems ranged from 0.9% to 29.3% in those studies (Figure 2). Subgroup analyses were conducted based on ICU type, geographical region, and publication year. The proportion of ENI frequency due to feeding tube problems was 3.7% in mixed/general ICU, 7.4% in surgical ICU and 14.1% in medical ICU. Studies published in 2014 or earlier reported the proportion of 11.1%, while those published in 2015 or later reported 7.9%. By geographic region, the proportion was 14.6% in Europe, 2.6% in Asia and 10.4% in the Americas. Additionally, four studies (23, 30, 32, 44) documented a total interruption duration of 11037.2 h, with 1276.0 h caused by feeding tube problems, representing 11.6% of the total ENI duration. The proportion of ENI duration due to feeding tube problems ranged from 1.3% to 25.6% in those studies (Figure 3).

In the study by Onuk et al. (19), the median ENI duration was 960 min during the ICU stay, with feeding tube problems resulting in the longest interruption, lasting 1,230 min. Similarly, O’Meara et al. (23) found that feeding tube problems accounted for 17.3% of the total ENI frequency and 25.6% of the total ENI duration, respectively.

#### 3.3.4 Hemodynamic instability

Among the 28 studies reviewed, 12 identified hemodynamic instability as a factor influencing ENI (14, 17, 19, 23, 26, 27, 29–32, 36, 42). Eleven studies (14, 17, 19, 23, 26, 27, 29–32, 36) reported 2,946 interruptions, with 116 episodes attributed specifically to hemodynamic instability, accounting for 3.9% of the total ENI frequency on average. The proportion of ENI frequency due to hemodynamic instability ranged from 0.9% to 20.0% in those studies (Figure 2). Subgroup analyses were conducted based on ICU type, geographical region, and publication year. The proportion of ENI frequency due to hemodynamic instability was 3.1% in mixed/general ICU, 7.1% in surgical ICU and 4.7% in medical ICU. Studies published in 2014 or earlier reported the proportion of 2.5%, while those published in 2015 or later reported 5.6%. By geographic region, the proportion was 10.2% in Europe, 1.6% in Asia and 3.4% in the Americas. Additionally, three studies (23, 30, 32) documented a total interruption duration of 7988.2 h, with 204.9 h attributed to hemodynamic instability, representing 2.6% of the total ENI duration. The proportion of ENI duration due to hemodynamic instability ranged from 1.1% to 5.1% in those studies (Figure 3).

In a prospective study by Salciute-Simene et al. (17), 73 critically ill patients experienced a total of 26 episodes of ENI (20.0%) due to hemodynamic instability. Similarly, Elpern et al. (42) reported that hemodynamic instability accounted for 13.5% of the total ENI duration.

## 4 Discussion

### 4.1 The current status of ENI

The review showed that the incidence and frequency of ENI was high, and ENI duration was prolonged in critically ill patients. The result was consistent with the studies by Liu et al. (48), indicating that approximately half of the patients experienced ENI, and EN was interrupted on 35% of the trial days.

However, the incidence (4.7%–100.0%), frequency (1–7 episodes during ICU stay), and duration (24.3–46.6 h during ICU stay) of ENI varied widely in critically ill patients. This variability can be attributed to inconsistencies in how ENI is defined across studies, particularly regarding interruption duration, which affects the comparability of results. For instance, O’Leary-Kelley et al. (22) define ENI as interruptions lasting over 15 min, while Salciute-Simene et al. (17) set the threshold at 1 h. A 15 min threshold captures shorter ENI events, such as repositioning or suctioning, that may not significantly impact nutrition intake, thereby increasing ENI incidence, frequency, and duration. Conversely, a 1 h threshold focuses on interruptions that disrupt nutritional delivery, such as those caused by surgeries, while overlooking brief ENI, resulting in lower reported incidence, frequency, and duration. Additionally, the clinical status of critically ill patients and the feeding route used can influence ENI. Severely ill patients, due to their condition or acute changes, often undergo procedures that extend ENI (47). Moreover, gastric feeding is more prone to complications such as gastric residuals, reflux, and vomiting, resulting in longer and more frequent ENI compared to post-pyloric feeding (48). To reduce this heterogeneity, future studies should standardize the definition of ENI, focusing on a patient-centered approach and establishing a consistent threshold for ENI duration and frequency. Additionally, uniform inclusion and exclusion criteria should be adopted across studies to ensure a more accurate assessment of ENI’s true impact on critically ill patients.

Subgroup analysis revealed that the incidence of ENI was higher in mixed/general ICU, studies published in 2014 or earlier and Oceania compared to other subgroups. This disparity may stem from the integration of medical and surgical ENI factors in mixed/general ICU. Meanwhile, the continuous advancement of medical technology and the growing emphasis placed by healthcare professionals on the management of ENI in critically ill patients may potentially reduce the incidence risk of ENI (49, 50). Notably, the high incidence in Oceania might be attributed to limited sample size (only one study included), which could introduce statistical power insufficiency as a potential bias.

### 4.2 Influencing factors of ENI

#### 4.2.1 Procedures

The results showed that procedures were the primary factor influencing ENI among critically ill patients, with airway procedures being the most common cause. Furthermore, procedural factors consistently accounted for a relatively large proportion across all subgroup analyses, which may be due to the advancements in medical technology since 2015 have increased the frequency of examinations and invasive procedures, further elevating ENI risks. Consistent with this finding, a prospective observational study by Lee et al. (30) had reported that airway procedures accounted for the highest frequency and duration of ENI.

Current clinical practices for pre-procedural feeding interruption in critically ill patients are primarily extrapolated from perioperative fasting guidelines designed for elective surgical patients, with no unified practice standard. The 2017 Practice Guidelines for Preoperative Fasting by the American Society of Anesthesiologists (ASA) recommend discontinuing liquid intake 6 h prior to procedures to reduce the risk of pulmonary aspiration in critically ill patients (51). In addition, in critically ill patients undergoing endotracheal intubation, diagnostic, or therapeutic procedures, anesthetic agents reduce respiratory muscle strength, inducing respiratory depression (52). Concurrently, certain pneumoperitoneum surgery may elevate intra-abdominal pressure (IAP), then transmitting through the diaphragm to increase intrathoracic pressure, which reduces pulmonary compliance and exacerbates respiratory depression (53, 54). In this context, continuous EN may elevates gastric pressure, which is associated with elevated IAP. Elevated IAP may impair respiratory mechanics (55, 56). Therefore, interrupting EN may improving respiratory depression. Moreover, the updated 2023 ASA guidelines further specify that clear liquids should be withheld for at least 2 h before anesthesia or procedural sedation (57). In light of these recommendations, ICU staff could carefully coordinate medical procedures to minimize unnecessary enteral nutrition interruptions and ensure both nutritional adequacy and patient safety.

#### 4.2.2 Gastrointestinal events

The result showed that gastrointestinal events had a slightly lower impact on the occurrence of ENI in critically ill patients compared to procedural factors, with GRV being the most common factor. Kim et al. (28) showed that gastrointestinal events were the main contributors to ENI, accounting for about 60% of all ENI frequency. That may be attributed to the inclusion of critically ill patients with contraindications to enteral nutrition, many of whom had pre-existing gastrointestinal symptoms, such as intestinal obstruction or gastrointestinal bleeding, at the time of enrollment.

According to the 2023 international guidelines (4) and supporting evidence from clinical studies such as Salciute-Simene et al. (17), temporary ENI is recommended for critically ill patients experiencing gastrointestinal intolerance, including high GRV, nausea/vomiting, or diarrhea. The reason for ENI caused by high GRV lies in its association with delayed gastric emptying and increased risk of aspiration. However, discrepancies exist in GRV thresholds for ENI across studies: while the guidelines define GRV ≥ 500 mL/6 h as the critical threshold (4), study has shown that higher GRV ranges (250–500 mL) do not statistically correlate with adverse outcomes such as aspiration pneumonia (58, 59). Hence, for critically ill patients with GRV < 500 mL, post-pyloric feeding may be prioritized. If symptoms persist, prokinetic agents could be considered. For issues that cannot be resolved by prokinetic agents or repositioning the feeding tube, short-term ENI may be used as an emergency measure (59). For diarrhea management, the guidelines advocate initial etiological evaluation and EN regimen optimization, Persistent diarrhea may benefit from probiotic-supplemented formulas to restore gut microbiota balance (59, 60). For high-aspiration-risk patients, post-pyloric feeding via nasojejunal tubes may be preferred, reducing reflux episodes compared to gastric feeding (4, 61).

#### 4.2.3 Feeding tube problems

The impact of feeding tube problems on ENI was slightly less significant than that of procedural and gastrointestinal event factors. Similarly, Sevda et al. (19), Nieuwkoop et al. (26) also reported that the proportion of ENI caused by feeding tube problems was relatively low compared to those caused by procedures and gastrointestinal events.

Feeding tube problems such as tube blockage, dislodgement or kinking may hinder nutrient delivery. In such situations, temporarily ENI allows for tube replacement or repositioning, which may prevent further gastrointestinal injury. To minimize ENI caused by feeding tube problems, regular monitoring, proper fixation and routine flushing protocols are essential. These measures may help maintain tube patency and ensure uninterrupted, effective nutritional support.

#### 4.2.4 Hemodynamic instability

Although the frequency and duration of ENI caused by hemodynamic instability was relatively low, its impact should not be underestimated. Salciute-Simene et al. (17) Showed that hemodynamic instability was a major factor in ENI, contributing the highest proportion. This is likely due to the severe nature of the patients’ conditions in this trial, with many suffering from comorbidities including 72.0% critically ill patients having septic shock and 36.6% requiring vasopressors.

In critically ill patients with hemodynamic instability, particularly those in shock or receiving vasopressors (NE ≥ 0.1 μg/kg/min) (62), patients with NE ≥ 0.1 μg/kg/min exhibit significantly lower citrulline levels (< 10 μmol/L) and elevated intestinal fatty acid-binding protein (I-FABP > 150 pg/mL), indicating mucosal ischemia (63, 64). Continuing EN may further compromise gastrointestinal blood flow, worsen ischemia, and increase the risk of complications such as non-occlusive mesenteric ischemia or intestinal necrosis (65). Therefore, to minimize further gastrointestinal damage with hemodynamic instability, it is advisable to temporarily interrupt EN when vasopressors (NE ≥ 0.1 μg/kg/min) are used, as this can exacerbate mucosal ischemia. Once hemodynamic stability is restored or the vasopressor dose reduced (NE < 0.05 μg/kg/min), EN may be cautiously reintroduced, starting with a low dose to ensure safe and effective delivery.

### 4.3 Implications

This study systematically evaluated 28 studies to identify four influencing factors of ENI: procedures, gastrointestinal events, feeding tube problems and hemodynamic instability. Early recognition of these factors enables targeted interventions to minimize unnecessary ENI. Specifically, ICU staff should adhere to evidence-based guidelines to minimize preoperative fasting durations. When gastrointestinal events occur, a tiered management approach should be implemented (e.g., adjusting infusion rates, modifying formulas and adding prokinetic agents). For feeding tube problems, establish protocols for regular monitoring, secure fixation and routine flushing. Additionally, critically ill patients with hemodynamic instability initiate low dose EN only after hemodynamic stabilization (e.g., NE ≤ 0.05 μg/kg/min). These measures may prevent ENI or reduce unnecessary ENI duration.

### 4.4 Limitations

The systematic review also has several limitations. This study only included English-language literature. This study was dedicated to conducting a comprehensive systematic review, meticulously examining the occurrence and determinants of ENI in ICU patients to provide valuable insights and foster improved patient care strategies. Furthermore, due to the heterogeneity in definitions across the included studies, we did not conduct a meta-analysis. The variations in how ENI was defined and measured across studies precluded a quantitative synthesis of the data. Instead, we conducted a descriptive systematic review, which limits the ability to draw definitive conclusions. In addition, the lack of a standardized definition for high volume of gastric residue, reflux and diarrhea across the included studies further constrained the comparability of this outcome. Therefore, future studies should standardize those definitions and include research from a broader range of languages to improve the generalizability and robustness of the findings.

## 5 Conclusion

The incidence and frequency of ENI in critically ill patients are notably high, with interruptions often lasting for extended durations. Various factors, such as procedures, gastrointestinal events, feeding tube problems, and hemodynamic instability, significantly influence ENI occurrence.

## Data Availability

The raw data supporting the conclusions of this article will be made available by the authors, without undue reservation.
